# Comprehensive Evaluation of *Toxoplasma gondii* VEG and *Neospora caninum* LIV Genomes with Tachyzoite Stage Transcriptome and Proteome Defines Novel Transcript Features

**DOI:** 10.1371/journal.pone.0124473

**Published:** 2015-04-13

**Authors:** Abhinay Ramaprasad, Tobias Mourier, Raeece Naeem, Tareq B. Malas, Ehab Moussa, Aswini Panigrahi, Sarah J. Vermont, Thomas D. Otto, Jonathan Wastling, Arnab Pain

**Affiliations:** 1 Pathogen Genomics Laboratory, Biological and Environmental Sciences and Engineering (BESE) Division, King Abdullah University of Science and Technology (KAUST), Thuwal, Jeddah, Kingdom of Saudi Arabia; 2 Centre for GeoGenetics, Natural History Museum of Denmark, University of Copenhagen, Copenhagen, Denmark; 3 Bioscience Core Laboratory (BCL), King Abdullah University of Science and Technology (KAUST), Thuwal, Jeddah, Kingdom of Saudi Arabia; 4 Institute of Infection and Global Health and School of Veterinary Science, Faculty of Health and Life Sciences, University of Liverpool, Liverpool, Merseyside, United Kingdom; 5 Parasite Genomics Group, Wellcome Trust Sanger Institute (WTSI), Genome Campus, Hinxton, Cambridge, United Kingdom; University at Buffalo, UNITED STATES

## Abstract

*Toxoplasma gondii* is an important protozoan parasite that infects all warm-blooded animals and causes opportunistic infections in immuno-compromised humans. Its closest relative, *Neospora caninum*, is an important veterinary pathogen that causes spontaneous abortion in livestock. Comparative genomics of these two closely related coccidians has been of particular interest to identify genes that contribute to varied host cell specificity and disease. Here, we describe a manual evaluation of these genomes based on strand-specific RNA sequencing and shotgun proteomics from the invasive tachyzoite stages of these two parasites. We have corrected predicted structures of over one third of the previously annotated gene models and have annotated untranslated regions (UTRs) in over half of the predicted protein-coding genes. We observe distinctly long UTRs in both the organisms, almost four times longer than other model eukaryotes. We have also identified a putative set of cis-natural antisense transcripts (cis-NATs) and long intergenic non-coding RNAs (lincRNAs). We have significantly improved the annotation quality in these genomes that would serve as a manually curated dataset for *Toxoplasma* and *Neospora* research communities.

## Introduction

*Toxoplasma gondii* and *Neospora caninum* are closely related coccidians that belong to a diverse group of parasitic protozoans of the phylum Apicomplexa. *T*. *gondii* is a zoonotic intracellular parasite capable of infecting any warm-blooded vertebrate. Estimated to infect around one-third of the human population, *T*. *gondii* causes neonatal mortality in humans and veterinary hosts and opportunistic infections in immuno-compromised individuals [1]. Apart from being a significant pathogen in its own right, *T*. *gondii* has gained more attention as a laboratory model to study Apicomplexan biology because it can be easily propagated and is experimentally tractable [2]. *N*. *caninum*, though similar to *T*. *gondii* in morphology, has a narrow host range of primarily cattle and dogs and is the leading cause of abortion in cattle resulting in significant economic losses [3]. The difference in host range of these two closely related species (having highly conserved gene content) has been of particular interest in studying molecular mechanisms behind host specificity in Apicomplexans [4].

Whole genome sequencing has been performed for 17 *T*. *gondii* strains till date that are publicly available in ToxoDb (http://www.toxodb.org) [5]. Of these, three prototypic strains- GT1 (Type I), ME49 (Type II) and VEG (Type III) serve as annotated reference genomes and are widely used as a basis for *Toxoplasma* research worldwide. The genome of *N*. *caninum* Liverpool strain was sequenced and annotated recently [4]. Both *T*. *gondii* and *N*. *caninum* genomes are approximately 62 million basepairs (Mb), divided into 14 chromosomes and contain around 7,500 predicted protein-coding genes.

Primarily, automated gene prediction tools such as TigrScan, HMMGlimmer, TwinScan, SNAP and AUGUSTUS have been used for genome annotation in coccidian genomes [6–9]. These *ab initio* gene predictors use a variety of genome level information such as codon frequencies, GC content and a small set of curated gene structures as a ‘training set’ to predict coding sequences and exon-intron boundaries. However, such predictors suffer from several limitations that compromise the accuracy of the predicted gene models. Genome annotation is the fundamental resource that links the genome to relevant biological information ranging from sequence information such as coding sequences, gene exon-intron boundaries and untranslated regions to functional information such as transcriptional start sites (TSSs), coding and non-coding RNA transcripts, and alternative splicing. Therefore, it is imperative to improve the quality of genome annotation because gene models form the basis of future research in an organism and errors in them can compromise downstream analyses. With the advent of high-throughput RNA sequencing and proteomic technologies, several annotation pipelines such as JIGSAW [10], AUGUSTUS [11] and PASA [12] have been developed that use several lines of experimental evidence in addition to *ab initio* gene predictions to significantly improve accuracy of predicted gene models. In fact, during the course of this work, parallel efforts using such computational tools have significantly improved the assembly and annotation quality of *T*. *gondii* ME49 genome [5,13]. In a previous comparative analysis of the *N*. *caninum* and *T*. *gondii* genomes [4], we have manually validated the organism–specific genes using tachyzoite stage transcriptomes (RNA-seq). However, there was no systematic effort to manually curate the predicted gene models using the RNA-seq datasets. Systematic manual curation of the gene models through careful inspection of the predicted gene structures in the light of several layers of experimental evidence (e.g. strand-specific RNA-seq, mass-spectrometry) leads to highly accurate gene models. Towards this goal of enriching and improving the genome annotation of these two Apicomplexan parasites, we resequenced the genomes of *Toxoplasma gondii* VEG strain (*Tg*VEG) and *Neospora caninum* LIV strain (*Nc*LIV) to close gaps and correct erroneous base-calls in the genome assembly. We performed strand-specific RNA sequencing (ssRNA-seq) and shotgun proteomics to get transcript and protein level evidences for curating gene models and predicting untranslated regions. Further, we annotated a set of putative natural antisense transcripts (cis-NATs) and long intergenic non-coding RNAs (lincRNAs) based on ssRNA-seq.

## Materials and Methods

### Parasite cultivation

Tachyzoites of *Toxoplasma gondii* VEG and *Neospora caninum* LIV strains were maintained in confluent layers of African Green monkey kidney cells (Vero) (ECACC, Salisbury, UK). The tachyzoites were harvested 3 or 4 days post-infection as described previously [14].

### Genome Sequencing

Genomic DNA was sheared using Covaris-E series. The sheared fragments were made into libraries following Truseq DNA LT kit protocol and size-selected in agarose gel for 200–300 bp insert size. Sequencing of the DNA libraries was done on an Illumina HiSeq 2000 with paired-end 100bp read chemistry.

### Genome improvement

Illumina reads were re-assembled with *T*. *gondii* VEG and *N*. *caninum* LIV genomes (ToxoDb v.8.0) as references. Sequence gaps were filled using Gapfiller (v1.10) [15] run for two 7 iterations with *k-mer* size set to 31. Single base errors and indels were corrected using iCORN [16] run for 5 iterations. The assemblies were evaluated by re-mapping reads onto the original and new assemblies and running REAPR [17] to compare the number of perfectly mapped reads. Gene annotations were transferred to the new assemblies using RATT [18] with “Transfer type” parameter as “Strain” (contains predefined nucmer parameters set for determining synteny between strains with 95–99% genome similarity). The *Tg*VEG and *Nc*LIV reads alignment bam files (ENA run IDs- ERR701180 and ERR701181) are available from the European Nucleotide Archive (ENA; http://www.ebi.ac.uk/ena/).

### Strand-specific RNA sequencing

Total RNA was extracted from day 3 and day 4 tachyzoite stage parasites using TRIzol method as described previously in [14]. Strand-specific RNA sequencing was performed from total RNA using TruSeq Stranded mRNA Sample Prep Kit LT according to manufacturer's instructions. Briefly, polyA+ RNA was purified from total RNA using oligo-dT dynabead selection. 1st strand cDNA was synthesized using randomly primed oligos followed by 2nd strand synthesis where dUTPs were incorporated to achieve strand-specificity. The cDNA was adapter-ligated and the libraries amplified by PCR. Libraries were sequenced on an Illumina HiSeq 2000 with paired-end 100bp read chemistry. The raw reads are available in ENA (accession numbers- ERR690605, ERR690606, ERR690607 and ERR690608).

Raw reads were aligned onto the genomes using TopHat version 2.0.9 [19] and transcripts were assembled using Cufflinks version 2.1.1 [20].

### Whole cell proteome analysis

Tachyzoite proteins were denatured with SDS, resolved in a 1-D polyacrylamide gel and in-gel tryptic digested as described in [21]. LC-MS/MS analysis of the digested peptides was carried out by Proxeon EASY-nLC unit (Bruker Daltonik, Germany) through a capillary column (0.1 × 150 mm, with C18 AQ of 3 μm particles and 200 pore size, Michrom42 BioResources) with a 60 min gradient of 2−40% solvent B (solvent A: 100% H_2_O; solvent B: 90% ACN, 0.1% formic acid). This system was connected to a LTQ-Orbitrap Velos (Thermo Scientific, Germany). Elution occurred at a flow rate of 500 nl/min and ionization was performed using an applied voltage of 1.5 kV to the emitter. Data were acquired using Xcalibur software in data- dependent mode to facilitate automatic switching between MS and MS2. The precursor ion scan MS spectra (m/z 300−1600) were acquired in the Orbitrap with resolution R = 60,000 at m/z 400 with the number of accumulated ions being 1 × 10^5^. The ten most intense ions were isolated and fragmented in the linear ion trap (number of accumulated ions; 10^5^). The resulting fragment ions were recorded in the Orbitrap with resolution R 15,000 at m/z 400. The lock mass option enabled accurate mass measurements in both MS and MS/MS mode. The poly-dimethylcyclosiloxane ions generated in the electrospray process from ambient air (protonated (Si(CH_3_)_2_O)_6_, m/z 445.120025) were used for internal recalibration in real time. In data-dependent LC-MS/MS experiments dynamic exclusion was used with 45 sec exclusion duration.

Tandem mass spectra were extracted by Xcalibur software. All MS/MS samples were analyzed using Mascot (Matrix Science, London, UK; version 2.4.0) against a concatenated organism-specific target-decoy database of i) protein-coding sequences; ii) protein sequences of all possible ORFs longer than 30 amino acids in all six reading frames of the genome and iii) protein-coding sequences of *Macaca mullata* (Rhesus monkey) to eliminate host cell contaminant peptides. Scaffold (version Scaffold_3.6.2, Proteome Software Inc., Portland, OR) was used to validate MS/MS based peptide and protein identifications. Peptide identifications were accepted if they could be established at greater than 95.0% probability as specified by the Peptide Prophet algorithm [19]. Protein identifications were accepted if they could be established at greater than 99.0% probability and contained at least 1 uniquely identified peptide. Protein probabilities were assigned by Protein Prophet algorithm [20]. The identified peptides were mapped onto the genomes using TBLASTN (exact matches). Identification and mapping were repeated again after manual curation to determine the final set of protein identifications.

### Manual curation, UTRs and long non-coding RNA prediction

Gene models and different tracks of evidence were visualized and curated in Artemis [22] genome visualization tool and Artemis Comparison Tool [23]. We curated the gene models in both the genomes based on three tracks of evidence in the following order of importance—1) Transcript evidence from ssRNA-seq, 2) Peptide evidence from mass spectrometry based shotgun proteomics and 3) protein level sequence similarity (TBLASTX) between *T*. *gondii* and *N*. *caninum* genomes (S1 Fig). Our manual curated gene models can be broadly classified into five categories-

1. Corrected gene models- We visually compared each gene model in both the genomes with the mapped ssRNA-seq paired-end reads. We modified only those gene models that had clear transcript-level evidence of exonic and intronic regions. We corrected exon-intron boundaries (defined by AT and GT/GC start and end sites respectively), added exons that were missing in transcript-level exonic regions, deleted exons that were present in transcript-level intronic regions and extended or truncated gene boundaries. These corrections also included exons that were wrongly predicted based on transcript reads on the opposite strand. Since ssRNA-seq cannot resolve translation start sites, we encountered cases where i) more than one frame of translation was possible for a gene, and ii) multiple transcriptional start sites were possible due to more than one start codon in the same reading frame. We were able to resolve these ambiguities in genes with peptide level evidence. By mapping peptide sequences, we were able to pinpoint the correct frame of translation and regions where genes should be extended to an upstream start codon. In other cases, the foremost start codon within the transcript was chosen as the translational start site.
2. Merged gene models- If a continuous transcript spanned across two or more predicted gene models with clear evidence of splice junctions between them, we identified them as incomplete gene models and merged them into a single gene.
3. Split gene models- In some gene models, it was evident that there were two or more separate neighboring transcripts from distinct lack of reads or splice junctions between them. We split and corrected them into separate genes.
4. New gene models- We predicted new genes in ORFs that had clear RNA-seq evidence and some of the highly expressed new genes also showed peptide evidence.
5. Deleted gene models- We considered a gene as spurious if it lacked RNA-seq evidence and overlapped with another RNA-seq supported curated gene, in the same or opposite strand. We did not delete non-overlapping genes that lacked RNA-seq evidence, as our RNA-seq data is only from tachyzoite stage and these genes might be expressed in other life stages and hence cannot be confidently called "spurious".

We also compared the gene models of orthologs in both the genomes during the curation process. This particularly helped in correcting genes with low or incomplete RNA-seq evidence and improved our confidence in newly created genes that had an ortholog in *T*. *gondii* or *N*. *caninum*.

To assess annotation quality, amino acid sequences of the publicly available set and our curated set of gene models were scanned for known protein domains using InterProScan v4.6 [24]. Full-length transcripts were assembled using Cufflinks version 2.1.1 [20] with default parameters. UTRs were predicted by comparing these transcripts and manually curated gene models using in-house Perl scripts. For each gene, the regions between start/stop of the transcript model and the start/stop of the manually curated gene model were inferred as UTRs. Polyadenylated non-coding RNA transcripts were predicted by adapting the protocol described in [25] and the non-coding potential of the transcripts were calculated using Coding Potential Calculator [26]. We assembled novel transcripts from the mapped RNA-seq reads using Cufflinks version 2.1.1 [20] and compared with the manually annotated gene models using cuffcompare [20]. Putative lincRNA transcripts were defined as those transcripts i) >200 base pairs long; ii) with coding potential score (calculated by CPC) lesser than −1; and iii) not overlapping with coding regions or UTRs in the same strand. Of these, transcripts that overlapped with gene models in the opposite strand with overlap region at least 100 bps were classified as anti-sense non-coding RNA (ancRNA) while the rest were classified as lincRNA. Candidates satisfying these criteria were then manually checked and only those that had uniform and distinct transcript evidence were retained.

The improved sequences and annotations of *T*. *gondii* VEG and *N*. *caninum* LIV can be found at ENA *(*Accession numbers between LN714489 and LN714514 for *Tg*VEG and between LN714474 and LN714488 for *Nc*LIV sequence and annotation).

## Results and Discussion

### Genome improvement and manual evaluation of gene models

**Fig 1:**
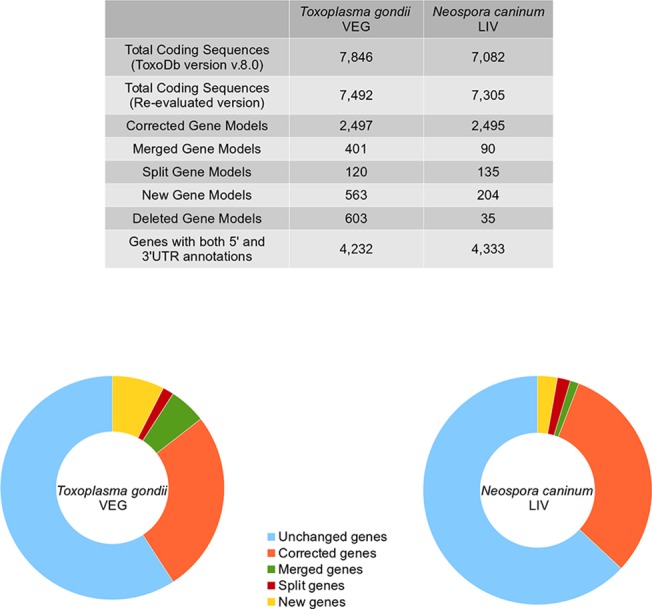
Summary of the manually curation of *Tg*VEG and *Nc*LIV (ToxoDb v8.0) genes. A gene model was “corrected” by adding/deleting exons or altering their exon-intron boundaries to conform to the transcript and peptide evidence. The corrected genes also include the models that were either “split” into two separate genes or “merged” into a single gene based on transcript splice-site evidence. “New” genes were annotated in open reading frames with clear expression evidence. Genes that lacked expression evidence and overlapped with an expressed gene model were considered spurious and “deleted”.

We improved the genome sequences by closing 111 and 39 gaps respectively in *Tg*VEG and *Nc*LIV nuclear genomes (S1 Table). We added 25,781 and 13,574 nucleotides in *Tg*VEG and *Nc*LIV genome assemblies respectively. Closure of gaps within genes and at exon-intron boundaries improved RNA-seq read mapping at these loci. This helped us identify incorrect splice sites and frame-shifts giving rise to erroneous gene models (S2B Fig). Then we manually verified the gene models of more than 7,000 genes each in *Tg*VEG and *Nc*LIV genomes, with RNA-seq and peptide evidence. We corrected 2,497 and 2,495 genes in *Tg*VEG and *Nc*LIV respectively, accounting for almost one-third of the genes. 401 *Tg*VEG and 90 *Nc*LIV genes were merged into a single gene, and 120 *Tg*VEG and 135 *Nc*LIV genes were split into two or three genes. A total of 560 and 200 new genes were created in *Tg*VEG and *Nc*LIV respectively while 603 and 35 genes were found spurious and deleted (Fig 1).

### Mass spectrometry and proteogenomic annotation

Controlling the target-decoy false discovery rate (FDR) at 5% and filtering for proteins with at least one unique peptide, we identified a total of 2,142 *Tg*VEG proteins with an estimated target-decoy FDR of 0.1% and 0.5% at the peptide and protein level respectively. Similarly, we identified 1,956 proteins in *Nc*LIV, albeit with a higher estimated peptide and protein FDR of 0.4% and 4.9%. Though our criterion for protein identification was at least one uniquely identified peptide, we observed that only 10% of the proteins identified were based on single peptide evidence with the rest having at least two or more peptides assigned to them. Also, we observed a good representation of *Tg*VEG-*Nc*LIV orthologs in the dataset, with 1,513 orthologous genes having peptide evidence in both the organisms, which proved useful for effective proteogenomic annotation. We mapped 16,737 and 14,777 unique identified peptides (exact matches) onto *Tg*VEG and *Nc*LIV genomes respectively and these consisted of either peptides mapping to protein-coding sequences or peptides mapping only to six-frame translated ORFs and not to annotated protein-coding regions (called “orphan” peptides). We were able to correct 13 *Tg*VEG and 30 *Nc*LIV gene models and create 16 *Tg*VEG and 25 *Nc*LIV new genes based on these “orphan” peptides that are also supported by RNA-seq evidence (S2A Fig). After our manual curation, only 22 *Tg*VEG and 33 *Nc*LIV “orphan” peptides still remained unaccounted for in the genomes. Of these, 8 *Tg*VEG and 16 *Nc*LIV peptides were mapping to intergenic regions while the rest emerged from intronic regions, being possible products of alternate splicing events. Our proteomics data provides mass spectrometry evidence for 51 additional *T*. *gondii* proteins that are currently available in ToxoDb v8.0.

The importance of integrating proteome data in genome annotation of *T*. *gondii* was clearly shown in a multi-platform global proteomic analysis [21], where 15% of the peptides mapped to alternate gene models and marked discrepancies in the annotated gene models such as wrong splice junctions and wrong frames of translation. We corrected similar discrepancies in our curation process reducing the final “orphan” peptides to just 0.001% of the ~15,000 total peptides in each genome. Peptide evidence was crucial to determine correct reading frames, translational start sites and to confirm correct splice junctions, particularly under circumstances where RNA-seq evidence was unclear.

### Annotation quality assessment

**Fig 2:**
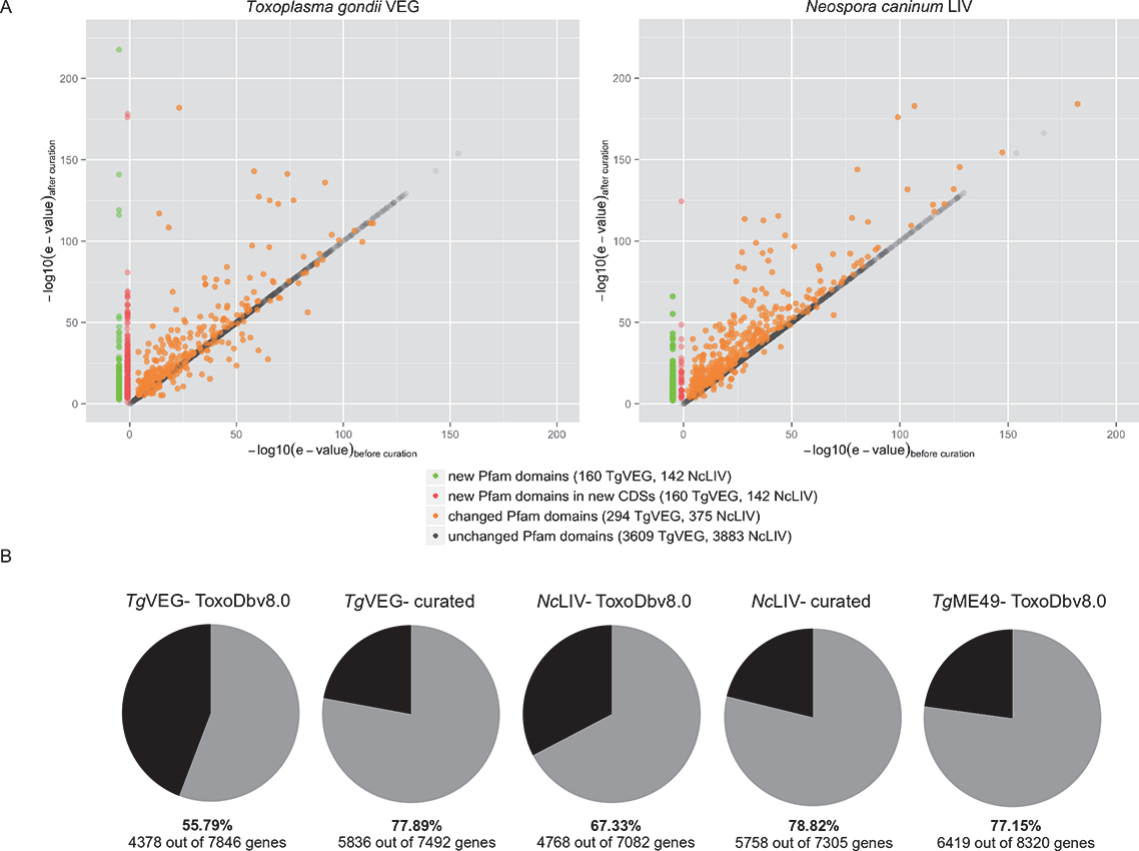
Qualitative and quantitative assessment of manually re-evaluated genomes. (A) We took confidence scores of Pfam domain hits (Pfam database v 26.0) as a rough indicator of the annotation quality and compared the e-value scores of Pfam domains before and after curation. Repetitive domains were omitted and the copy with the lowest e-value score is used for comparison. A 10-fold change in the e-value was considered as a significant change. While the e-values of Pfam domains in unchanged genes remain the same, we find a general increase in Pfam domain hit significance (decreasing e-values) after our curation (orange). We also find new Pfam domain hits appearing in the corrected genes (green) and in newly created genes (red). (B) Quantitative assessment of manual curation. Functional domain content of a genome is a crude indicator of annotation quality; therefore we compared the proportion of genes having a domain hit from InterProScan (grey) to genes without any domains (black). We find a ~20% increase in genes with functional domains after curation in *Tg*VEG and ~10% in *Nc*LIV genome.

We selected Pfam domain content and confidence scores of the domain hits as crude indicators of improvement in annotation quality. Tools such as InterProScan [24] can identify known domains present in a protein and the percentage of proteins in an annotated genome with domains can indicate the annotation quality. We observed a 22% and 12% increase in domain content in *T*. *gondii* and *N*. *caninum* respectively after our manual curation. We discovered 362 and 177 new domains respectively from Pfam database v26.0 [27] alone, of which 202 and 35 domains were present in newly created genes (Fig 2B).

Apart from the number of domain hits, the quality of these hits, reflected by their e-value scores, can also judge annotation quality. A correct gene model is expected to have better sequence alignment with a known functional domain than an incorrect model, gaining a lower e-value. In our set of corrected gene models, we recorded a greater than 10-fold decrease in e-values of Pfam matches in 223 and 349 domains in *T*. *gondii* VEG and *N*. *caninum* LIV respectively. In a small number of cases, we observed an increase in e-values because our curation led to insertions within the existing Pfam domains causing it to split into two or more domains with higher e-values. The general trend of increased confidence scores of the Pfam domains and the gain of new functional domains in the genomes together indicate a significant improvement in the annotation quality (Fig 2A). The percentage of protein-coding genes having functional domains stand as of now at 77.89% and 78.82% in *T*. *gondii* and *N*. *caninum* which is comparable to 77.15% in the recently improved annotation of Type II *T*. *gondii* ME49 (ToxoDb v8.0).

### *T*. *gondii* and *N*. *caninum* have characteristic long untranslated regions in transcripts

Based on assembled transcripts from our ssRNA-seq datasets, we annotated 4,711 5’UTRs and 4,523 3’UTRs in *Tg*VEG, with 4,232 *Tg*VEG genes gaining both 5’ and 3’UTR annotations. Similarly, we annotated 4,704 5’UTRs and 4,638 3’UTRs in *Nc*LIV, with 4,333 *Nc*LIV genes gaining both 5’ and 3’UTR annotations.

**Fig 3:**
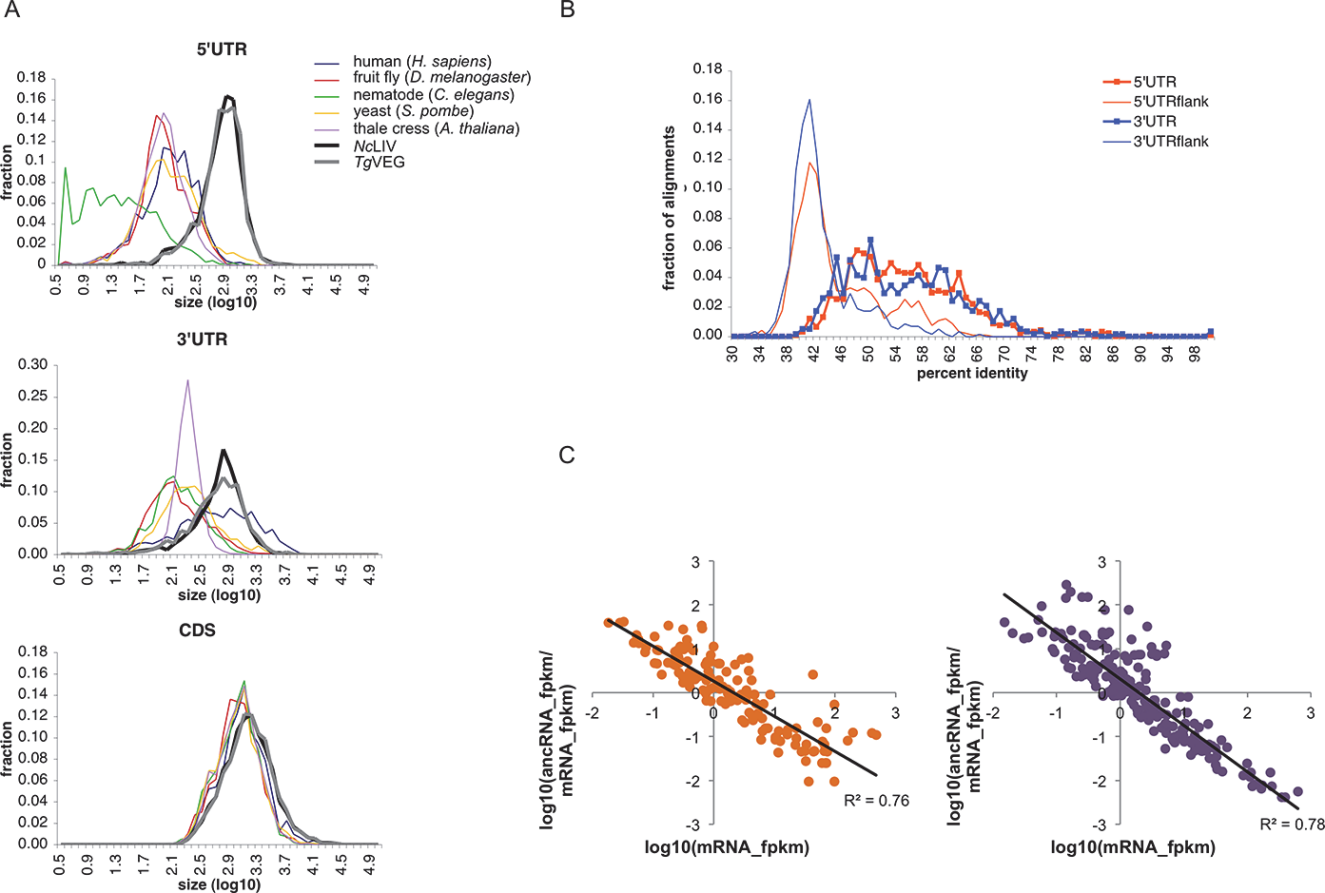
Long untranslated regions and putative anti-sense non-coding RNAs in *Tg*VEG and *Nc*LIV. (A) Length distribution of 5’UTRs, 3’UTRs and CDS in *Toxoplasma gondii*, *Neospora caninum*, *Schizosaccharomyces pombe*, *Arabidopsis thaliana*, *Caenorhabditis elegans*, *Drosophila melanogaster* and *Homo sapiens*. 5’UTRs are found to be strikingly large in the parasites, almost 4 times higher than other eukaryotes. 3’UTRs are comparable to those in human and longer than other eukaryotes. (B) Sequence conservation across UTRs and their flanking intergenic regions. UTR regions are generally more conserved than their flanking intergenic regions. (C) Log abundance ratio of antisense non-coding RNA (ancRNA) and sense coding mRNA pair versus sense coding RNA. There is an inverse relation between abundances of ancRNA and their sense mRNA counterpart.

We compared the UTR lengths with those in other model eukaryotes, namely *Schizosaccharomyces pombe* (genes were retrieved from PomBase, http://www.pombase.org/), *Arabidopsis thaliana*, *Caenorhabditis elegans*, *Drosophila melanogaster* and *Homo sapiens* (RefSeq gene annotations of all four model organisms were retrieved from UCSC Genome Browser, http://www.genome.ucsc.edu/). We observed that the 5’UTRs are strikingly longer in *T*. *gondii* and *N*. *caninum* (810–860 nucleotides) compared to other model eukaryotes compared in the study (Fig 3A). Mann-Whitney test proved a highly significant increase (all p-values less than 2.22 x 10–15, Bonferroni corrected, 10 tests) in 5’UTR lengths in *T*. *gondii* and *N*. *caninum* against the model eukaryotes. 3’UTRs were similar to those in humans but longer than those in other eukaryotes. We also compared the UTR lengths separately for genes conserved and not conserved across these eukaryotes to determine whether the differences in length distributions were arising mainly from species-specific genes. We found that the UTRs were uniformly longer in *T*. *gondii* and *N*. *caninum* than in other eukaryotes independent of gene conservation. Next, we checked whether UTR sizes differed between coccidian genes (orthologs in *T*. *gondii* and *N*. *caninum*) and non-coccidian genes (orthologs in *T*. *gondii*, *N*. *caninum* and *P*. *falciparum* (PlasmoDb v9.0)). No significant variation was seen between UTR lengths in genes restricted to coccidians and the set of genes that were also shared with *Plasmodium*.

To identify gene families that were enriched with long UTRs, known and predicted host-interaction genes such as rhoptry, microneme and dense granule genes, and apiAP2 family of transcription factors (apiAP2 Tfs) were retrieved from [4]. Comparing the UTR lengths of these gene classes with the median UTR size of all genes, we observed that apiAP2 transcription factors have significantly longer 5’ and 3’ UTRs in both *T*. *gondii* and *N*. *caninum* (Table 1). Of the 68 apiAP2 Tfs in each organism identified by [4], 46 TgVEG and 48 NcLIV genes had either or both of their UTRs annotated. Out of these, 37 *Tg*VEG and 41 *Nc*LIV apiAP2 Tfs had longer UTRs than the average UTR size in the respective genomes, and of which a majority of them were orthologs (28 apiAP2 Tfs ortholog pairs). In contrast, microneme proteins in *N*. *caninum* had significantly shorter 5’UTRs than rest of the genes. Next, we analysed size variation and sequence conservation between *T*. *gondii* and *N*. *caninum* UTRs to find whether the high level of conservation between their coding regions also extended to their UTRs. We observed that the UTR sizes were reasonably correlated between *Tg*VEG-*Nc*LIV orthologs, though less correlated as compared to the intron and coding region sizes. Kendall tau correlations were 0.54 and 0.38 (p-value less than 2.2 x 10^–16^) for 5’UTRs and 3’UTRs respectively while coding regions and introns had high size correlations of 0.95 and 0.87. For sequence conservation, we selected neighboring genes with annotated UTRs and with at least 1,000 basepairs between the adjacent UTRs. UTR sequences and 500 basepair flanking intergenic sequences were compared between *T*. *gondii* and *N*. *caninum* and the percent identity (defined as the number of identical positions in the alignment per base-pair of the shorter sequence of the pair) was calculated. The UTRs were generally more conserved between the species compared to the intergenic regions with a median percent identity of ~54% compared to ~40% in intergenic regions (Fig 3B).

**Table 1:** Comparison of UTR sizes of specific gene families in *T*. *gondii* VEG and *N*. *caninum* LIV.

Organism	Gene group	Number of genes with an annotated UTR	5'UTRs					3'UTRs				
			Median size	Average size	Number of UTRs larger than median UTR size in genome	Number of UTRs smaller than median UTR size in genome	Probability*	Median size	Average size	Number of UTRs larger than median UTR size in genome	Number of UTRs smaller than median UTR size in genome	Probability^a^
*Toxoplasma gondii VEG*	All genes	4232	857	969.2	———-	———-		741.5	934.5	———-	———-	
	Rhoptry proteins	30	926	1031.5	16	14		615	888.8	14	16	
	Microneme related	15	716	697.1	7	8		609	880.5	6	9	
	Dense granules	5	448	720.6	1	4		370	1224.6	2	3	
	AP2 transcription factors	46	1445	1644.3	37 (28)^b^	9	2 x 10^–5^	1490.5	1663.9	38 (28)	8	4.6 x 10^–6^
*Neospora caninum LIV*	All genes	4333	816	919.9				755	902.4			
	Rhoptry proteins	29	792	949.5	14	15		857	1006.9	16	13	
	Microneme related	13	392	485.2	2	11	0.011	653	781.9	6	7	
	Dense granules	4	271.5	630.2	1	3		279	338.8	1	3	
	AP2 transcription factors	48	1465.5	1644.3	41 (28)	7	3.1 x 10^–7^	1438	1591.4	41 (28)	7	3.1 x 10^–7^

The median sizes of UTRs of genes belonging to specific gene families that had an annotated UTR were calculated and compared against the median size of UTRs of all genes in the genomes that had an annotated UTR. Wherever a significant difference was found, the probability values have been shown. AP2 transcription factors have significantly longer UTRs than rest of the genes.

^a^ Probability of the identified UTRs having size higher/lower than the median size for all genes, just by chance assuming a binomial distribution.

^b^ Numbers within brackets are the number of *Tg*VEG-*Nc*LIV orthologs.

UTRs play an important role in post-transcriptional regulation of gene expression and modulate mRNA transport from the nucleus, their subcellular localization, translation efficiency and stability in eukaryotes [28]. Apicomplexan parasites have complex life cycles that consist of both proliferative and latent life stages, which enable the parasite to propagate through diverse vector and host environments. The parasites regulate the transition between these life-stages with high precision by regulating gene expression at multiple levels—epigenetic modifications, transcription and translation. The discovery of Pumilio (Puf) RNA-binding proteins in *Plasmodium* [29] and life stage-specific expression of elF4A RNA helicase in *Toxoplasma* [30] provides clear evidence that UTRs play a key role in post-transcriptional regulation in Apicomplexans. Puf proteins bind to 3’UTRs of specific gene transcripts to repress their translation and affect their stability. On the other hand, elF4A unwinds the secondary structure of 5’UTRs initiating translation of the mRNA transcripts in *Toxoplasma gondii*. Cis-acting elements in 5’ and 3’ UTRs play a key role in transcripts that undergo DDX-6 class RNA helicase DOZI mediated translational repression in *Plasmodium* female gametocytes [31]. Recently, both polysome profiling [32] and ribosome profiling [33] in *Plasmodium* have shown increased occupancy of RNA-binding proteins and ribosomes in 5’UTR regions but how this affects translation efficiency is still unclear. To further elucidate these regulatory mechanisms, annotation of UTRs in the genome is essential to discover binding motifs of RNA-binding proteins, target sites of regulatory non-coding RNAs and repetitive elements that affect translation efficiency. With the help of ssRNA-seq, we were able to annotate UTRs for over two-thirds of the genes and we found the 5’UTRs to be strikingly large in the parasites with a median size of 810–860 nucleotides, almost 4 times higher than other model eukaryotes, included in the study. The previous estimation of 5’UTR size in *Toxoplasma* was 288 nucleotides based on expressed sequence tags (EST) analysis [34] and 120–140 nucleotides based on transcriptional start site sequencing (TSS-seq) [35]. But these estimates were based on only a subset of genes. The EST data covered only 814 annotated genes and the estimate from TSS-seq was based on a subset of genes with signal peptides for higher reliability. In fact, when all the genes were taken into account along with the predicted TSS sites, the authors estimated a higher 5’UTR size in the range of 580–600 nts [35]. But they considered this estimate as unreliable, reasoning that the 5’ ends of the ORFs could be incorrect because of wrong gene annotations in *Toxoplasma* shown by previous studies. Since our UTR dataset was generated after manually curating the gene models and from strand-specific RNA-seq in two closely related organisms, we believe that the large size of 5’UTRs are not due to incorrect gene models but signify true biological features of these protein-coding genes. A recent study [33] reported similar findings of long 5’UTRs in the range of 607 to 1040 nts in *Plasmodium*. Therefore, long 5’ UTRs could be a common feature among Apicomplexan parasites playing a key role in their tightly regulated life cycles. Unusually long 5’UTRs in a subset of genes in *T*. *gondii* and *N*. *caninum* such as apiAP2 Tfs could have a possible role in gene regulation in these parasites and further studies are required to test this hypothesis.

We also mapped modified histone marks (H4ac, H3K9ac and H3K4me3) that are known activation marks, previously identified in *T*. *gondii* RH tachyzoites [36], onto *Tg*VEG and compared the regions to our annotated 5’UTRs. Out of the 52 high quality histone marks, 40 overlapped with the 5’UTRs, usually starting at a few hundred bases upstream of the 5’UTR and spanning the whole length or part of the 5’UTR (S3 Fig). The other 12 peak regions were near the 5’ end of gene models whose 5’UTRs were not annotated in our dataset due to lack of clear RNA-seq evidence. Taken together, we provide a high-confidence set of UTR annotations in both the parasite genomes.

### Putative non-coding RNA in *Toxoplasma gondii* and *Neospora caninum*

From a total of 1,270 and 1,940 polyadenylated, intergenic, non-coding transcripts assembled in *Tg*VEG and *Nc*LIV respectively, we identified 688 putative lincRNA and 209 putative ancRNA in *Tg*VEG; 881 lincRNA and 300 ancRNA in *Nc*LIV satisfying the criteria mentioned in the Methods. We observed similar average lengths of 1,310 and 1,369 nts for lincRNA in *Tg*VEG and *Nc*LIV respectively. The *Tg*VEG ancRNA were on an average 2,711 nts long with a mean overlap length of 1,056 nts with the sense coding gene while the *Nc*LIV ancRNA were 2,527 nts long with a mean overlap length of 924 nts. Both lincRNA (~72%) and ancRNA (~80%) species were predominantly unspliced transcripts in both the organisms, similar to previously reported in *Toxoplasma gondii* [13]. We also observe a strong anti-correlation (R-square value of ~0.77) between the expression of anti-sense non-coding RNA and sense coding mRNA in the sense-antisense transcript pairs (Fig 3C). Comparing ancRNA-mRNA pairs at 144 loci in *Tg*VEG and 213 loci in *Nc*LIV, we see that as the expression of a coding mRNA decreases, the expression of its corresponding antisense non-coding RNA transcript increases. Our results in *T*. *gondii* and *N*. *caninum* substantiate similar findings from previous studies using SAGE data in *T*. *gondii* [34] and *P*. *falciparum* [37]. This points to interesting regulatory functions for these ancRNA transcripts in Apicomplexan parasites that remain to be elucidated.

Long non-coding RNA have been shown to have roles in regulating transcription, post-transcriptional regulation and chromatin remodeling [38]. Though their precise cellular functions remain to be elucidated, many studies have discovered thousands of lncRNA genes in fruit fly [39], zebrafish [40] and roundworm [25]. lncRNA in parasites have not been yet investigated in depth though they have been shown to have specific functions in *Oxytricha trifallax* [41] and *Leishmania* [42], and many putative lncRNAs have been discovered in *Plasmodium falciparum* [37,43,44].

Here we have identified a set of putative lincRNA and ancRNA transcripts in *T*. *gondii* and *N*. *caninum*. It is worth noting that since we used poly(A)+ RNA-seq libraries, our dataset lacks non-polyadenylated lncRNA populations. lincRNA in *T*. *gondii* and *N*. *caninum* have a mean length of ~1,340 bps, are predominantly single exon transcripts and have similar A/U content as untranslated and intergenic regions. We also provide additional RNA-seq evidence for inverse correlation existing between anti-sense and sense transcript abundances found by [34] based on SAGE and extend the same phenomenon to *N*. *caninum*.

## Conclusions

High-throughput sequencing has enabled researchers to sequence the whole genome of several important parasites to gain unparalleled insights into their life cycle, biological mechanisms and evolution. The quality of genome annotation greatly influences any downstream study, where faulty or incomplete gene models alter the gene sequence and affect analyses based on them. Hence, efforts towards improving the annotation of the genome are beneficial for any genome-based research. Here, we have identified and manually corrected publicly available gene models in *T*. *gondii* VEG and *N*. *caninum* LIV genomes, which amounted to over one-third of the total predicted protein-coding genes. Apart from roughly 468 *Tg*VEG (6.2% of the genes) and 845 *Nc*LIV (11.6%) genes that had no RNA-seq evidence, the correctness of each gene model has been manually verified with transcript and peptide evidence. While we observe a significant increase in annotation quality after manual curation, the genomes may still contain a small fraction of inaccurate gene models that could not be resolved properly by our supporting datasets. Annotation is a continuous process and more lines of evidence in the future would help in improving the genome quality further. For example, transcriptome data across all life-stages rather than just the tachyzoite stage used here could provide better transcript evidence for curating stage-specific expressed genes or alternative splicing events. Similarly long range genomic reads generated from third generation sequencing technologies will help to provide much improved genome assemblies. Apart from accurate annotation of protein-coding genes, generating a catalog of other genomic features and potential regulatory factors such as non-coding RNA and untranslated regions makes a genome annotation more comprehensive. UTRs are gaining prominence in post-transcriptional regulatory processes and their role in Apicomplexan parasites with heavily regulated life cycles need to be further elucidated. We have created a comprehensive catalog of UTRs in *T*. *gondii* and *N*. *caninum* based on strand-specific RNA-seq. Both the parasites have uniquely long UTRs compared to other eukaryotes indicating UTRs may play key regulatory roles at least in a subset of genes in these parasites. This is further implied by the fact that apiAP2 transcription factors, a major family of transcriptional regulators have significantly longer UTRs than the rest of the genes. Long non-coding RNAs are yet another interesting class of transcripts with potential roles in transcriptional regulation in eukaryotes and we have identified a putative set of lncRNAs in both the parasites. With description of these catalogs and manually curated protein-coding genes, we have vastly improved the genome annotation of *Toxoplasma gondii* VEG and *Neospora caninum* LIV for future use by the research community.

## Supporting Information

S1 Fig ARTEMIS screenshot showing manual re-evaluation of chromosome XII in *Tg*VEG and *Nc*LIV. Manual re-evaluation was performed by overlaying multiple levels of evidence—ssRNA-seq (blue and green read stacks) viewed as aligned bam file, peptide sequences and TBLASTX hits between *Tg*VEG and *Nc*LIV (red bands). (A) A previously predicted gene is split into two separate genes (purple), a previously predicted gene model whose structure was corrected (orange). Previously predicted gene models are in blue and the spurious gene models that were deleted are marked in red. (B) Genes were merged (green) and a new gene was created (yellow) based on ssRNA-seq evidence.
(TIF)

S2 Fig Gap filling and peptide evidence help in refining gene models viewed in Artemis. (A) Mapped peptides (green) provide additional evidence to splice junctions and orphan peptides (grey) that do not fall entirely or partially within a CDS help in resolving the correct start codon for a gene. The 3’ end of a gene (blue) was corrected (orange) by extending to a start codon further upstream based on peptide evidence. (B) Filling gaps in the genome sequence aided in resolving the correct structure of a gene model. Top and bottom panel are the original and re-evaluated versions respectively. Region marked in red is the gap at the exon-intron boundary, making it difficult to identify the splice site, as RNA-seq reads did not map to the region. Gap closure resulted in a shift in the reading frame and remapping enabled us to resolve the splice site.
(TIF)

S3 Fig Histone peaks mapped onto *Tg*VEG chromosome Ib. The 52 histone peak regions were extracted from the 650 kbps region in *T*. *gondii* RH chromosome Ib and BLASTN was used to identify the similar regions in *Tg*VEG. Bottom panel shows the corresponding 650 kbp region in *Tg*VEG. Middle and top panel shows detailed view of the overlap between histone peaks and 5’UTRs with strand-specific RNA-seq coverage plot respectively. 40 histone peaks (green) overlap with 5’UTRs (orange) of genes (blue) and no 5’UTRs were annotated in the rest of the peaks (pink).
(TIF)

S1 Table Genome improvement by filling gaps and correcting bases in *Tg*VEG and *Nc*LIV genomes. (XLSX)

S2 Table Whole cell shotgun proteomics data for *Tg*VEG and *Nc*LIV from mass spectrometry. (XLSX)

S3 Table Comparison of our curated gene models with the present version of *Tg*VEG gene models ToxoDb v12.0. Similarity between our curated gene models and recently updated ToxoDb v12.0 *Tg*VEG genes were assessed by pairwise BLASTP.
(XLSX)
