# Supplementary figures and images for: Comprehensive Evaluation of Toxoplasma gondii VEG and Neospora caninum LIV Genomes with Tachyzoite Stage Transcriptome and Proteome Defines Novel Transcript Features
Source: PLoS One. 2015 Apr 13;10(4):e0124473. doi: 10.1371/journal.pone.0124473 (PMC4395442; doi:10.1371/journal.pone.0124473)

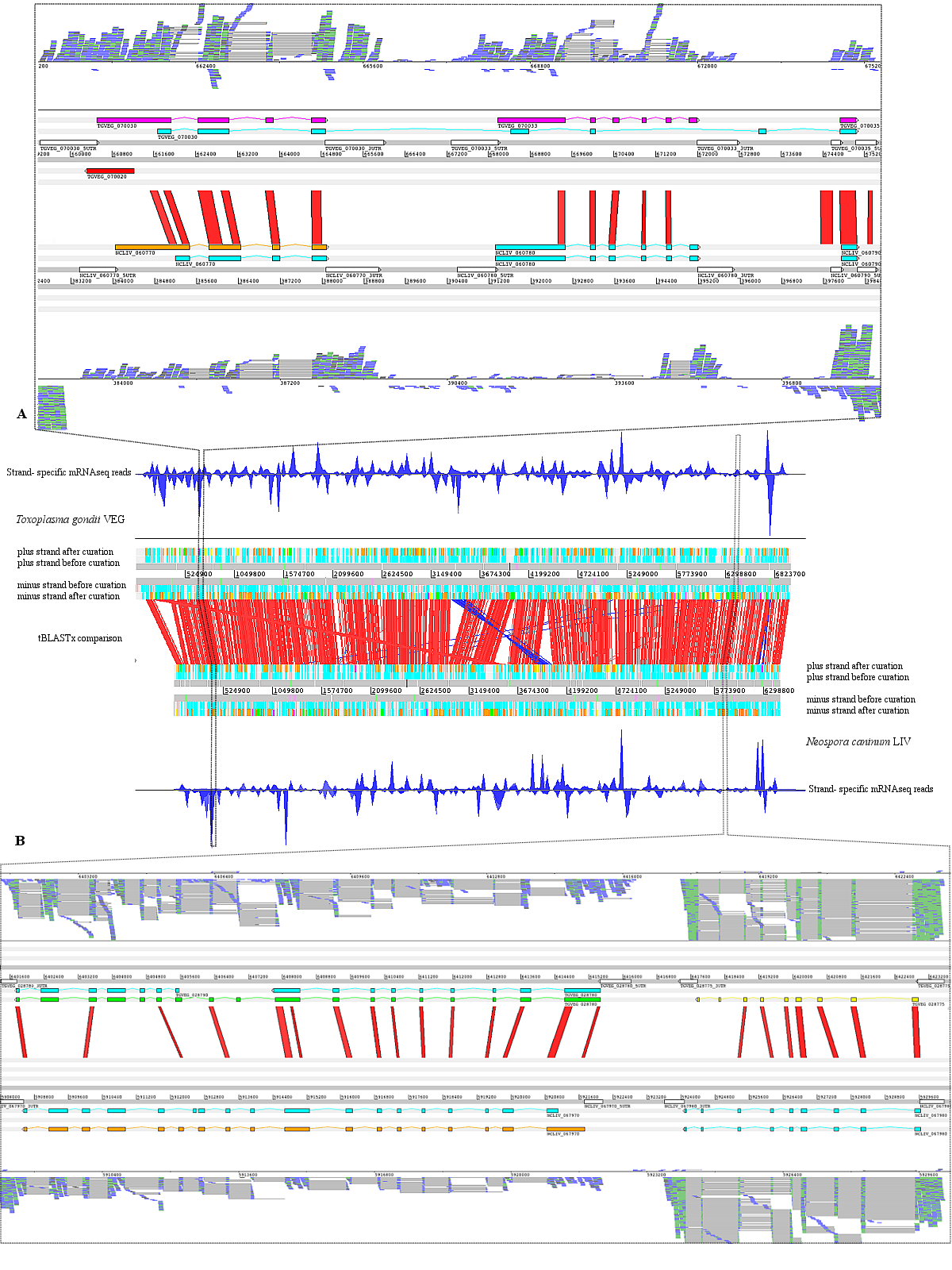

Supplement: S1 Fig — Manual re-evaluation was performed by overlaying multiple levels of evidence—ssRNA-seq (blue and green read stacks) viewed as aligned bam file, peptide sequences and TBLASTX hits between TgVEG and NcLIV (red bands). (A) A previously predicted gene is split into two separate genes (purple), a previously predicted gene model whose structure was corrected (orange). Previously predicted gene models are in blue and the spurious gene models that were deleted are marked in red. (B) Genes were merged (green) and a new gene was created (yellow) based on ssRNA-seq evidence. (TIF) [file pone.0124473.s001.tif]

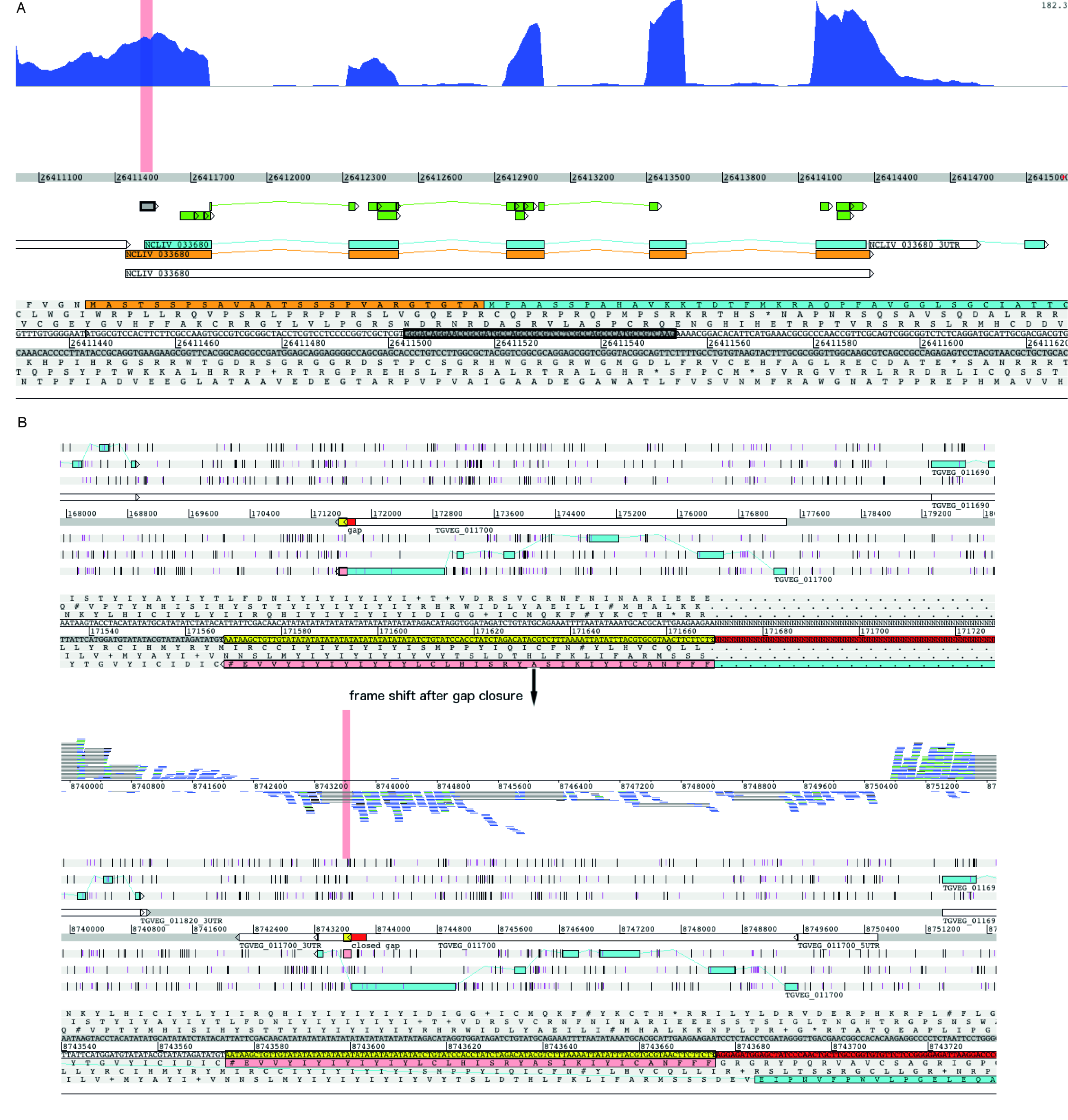

Supplement: S2 Fig — (A) Mapped peptides (green) provide additional evidence to splice junctions and orphan peptides (grey) that do not fall entirely or partially within a CDS help in resolving the correct start codon for a gene. The 3’ end of a gene (blue) was corrected (orange) by extending to a start codon further upstream based on peptide evidence. (B) Filling gaps in the genome sequence aided in resolving the correct structure of a gene model. Top and bottom panel are the original and re-evaluated versions respectively. Region marked in red is the gap at the exon-intron boundary, making it difficult to identify the splice site, as RNA-seq reads did not map to the region. Gap closure resulted in a shift in the reading frame and remapping enabled us to resolve the splice site. (TIF) [file pone.0124473.s002.tif]

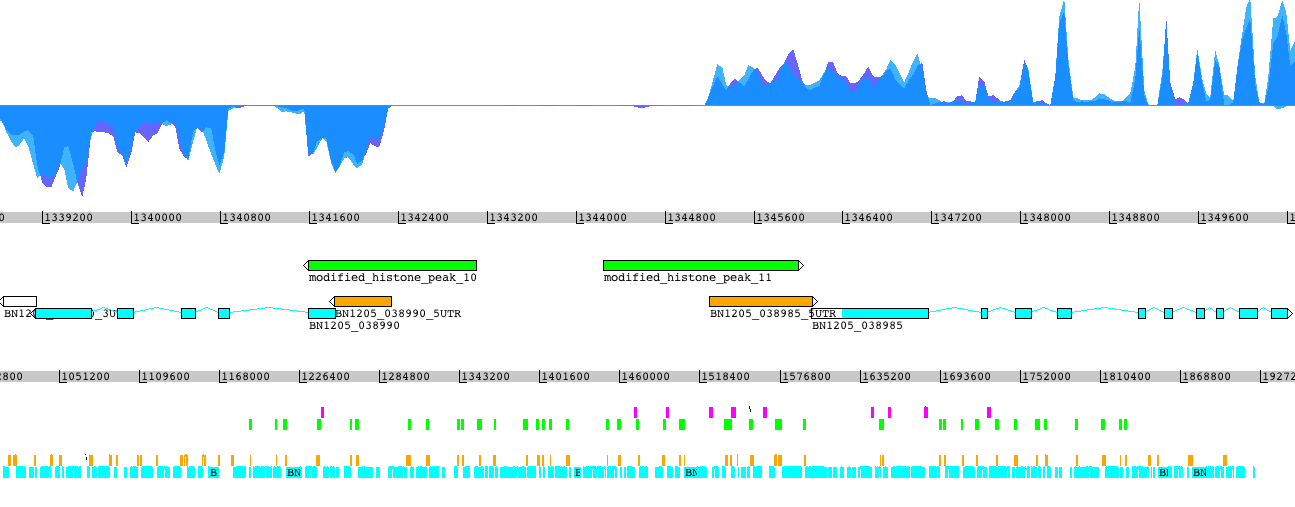

Supplement: S3 Fig — The 52 histone peak regions were extracted from the 650 kbps region in T. gondii RH chromosome Ib and BLASTN was used to identify the similar regions in TgVEG. Bottom panel shows the corresponding 650 kbp region in TgVEG. Middle and top panel shows detailed view of the overlap between histone peaks and 5’UTRs with strand-specific RNA-seq coverage plot respectively. 40 histone peaks (green) overlap with 5’UTRs (orange) of genes (blue) and no 5’UTRs were annotated in the rest of the peaks (pink). (TIF) [file pone.0124473.s003.tif]
